# Numerical Simulation of the Photobleaching Process in Laser-Induced Fluorescence Photobleaching Anemometer

**DOI:** 10.3390/mi12121592

**Published:** 2021-12-20

**Authors:** Yu Chen, Shuangshuang Meng, Kaige Wang, Jintao Bai, Wei Zhao

**Affiliations:** State Key Laboratory of Photon-Technology in Western China Energy, International Collaborative Center on Photoelectric Technology and Nano Functional Materials, Institute of Photonics & Photon Technology, Northwest University, Xi’an 710127, China; 201920590@stumail.nwu.edu.cn (Y.C.); 201931692@stumail.nwu.edu.cn (S.M.); wangkg@nwu.edu.cn (K.W.); baijt@nwu.edu.cn (J.B.)

**Keywords:** microfluidics, photobleaching process, laser-induced fluorescence photobleaching anemometer, super-resolution

## Abstract

At present, a novel flow diagnostic technique for micro/nanofluidics velocity measurement—laser-induced fluorescence photobleaching anemometer (LIFPA)—has been developed and successfully applied in broad areas, e.g., electrokinetic turbulence in micromixers and AC electroosmotic flow. Nevertheless, in previous investigations, to qualitatively reveal the dynamics of the photobleaching process of LIFPA, an approximation of uniform laser distribution was applied. This is different from the actual condition where the laser power density distribution is normally Gaussian. In this investigation, we numerically studied the photobleaching process of fluorescent dye in the laser focus region, according to the convection–diffusion reaction equation. The profiles of effective dye concentration and fluorescence were elucidated. The relationship between the commonly used photobleaching time constant obtained by experiments and the photochemical reaction coefficient is revealed. With the established model, we further discuss the effective spatial resolution of LIFPA and study the influence of the detection region of fluorescence on the performance of the LIFPA system. It is found that at sufficiently high excitation laser power density, LIFPA can even achieve a super-resolution that breaks the limit of optical diffraction. We hope the current investigation can reveal the photobleaching process of fluorescent dye under high laser power density illumination, to enhance our understanding of fluorescent dynamics and photochemistry and develop more powerful photobleaching-related flow diagnostic techniques.

## 1. Introduction

Laser-induced fluorescence photobleaching anemometer (LIFPA) is a velocity measurement technique developed by Wang in 2005 [1] and primarily for micro/nanofluidics. It measures flow velocity due to the photobleaching nature of fluorescent dye under a high laser power density. When fluorescent dye molecules are excited by a wavelength-matched laser, either the structures of molecules could be damaged due to the photo instability of the dye molecules [2], or the excited states could be quenched by dye–dye or solvent interactions, when the power density of the excitation beam exceeds a certain threshold. The photobleaching results in a decrease in the effective concentration (C) of fluorescent dye and a weakening fluorescent signal with increasing irradiation time [3]. The faster the fluorescent molecules pass through the excitation region in a flow field with uniform fluorescent molecules, the higher the effective concentration of fluorescent dye, and the higher the fluorescent intensity collected [4]. By establishing the monotonic relationship [5] between fluorescence intensity and flow velocity, the velocity of the flow field can be calculated by detecting the fluorescence signal intensity in the spot area [6]. This technique has been successfully applied to the velocity measurement of complex flow fields such as linearly and nonlinearly oscillating electroosmotic flow [7,8,9,10], and microelectrokinetic turbulence [11,12,13,14,15].

The dynamics of photobleaching is the kernel of LIFPA applications and determines the performance of the LIFPA system. Wang qualitatively analyzed the photobleaching process [1], attributing it to a first-order model of reaction:

(1) $$I_{f}=I_{f0}\times \exp\left(-t/\tau \right)=I_{f0}\times \exp\left(-d_{f}/U\tau \right)$$

where $I_{f}$ represents the overall fluorescence intensity of the laser focal area, $I_{f0}$ is the initial $I_{f}$ at t=0, t is the bleaching time that the fluorescent molecules pass through the region of laser focus diameter $d_{f}$, τ is the photobleaching time constant, U represents flow velocity. Then, Zhao et al. [16] studied the photobleaching process and established a theoretical formula to estimate the temporal resolution of LIFPA.

(2) $$I_{f}=d_{f}I_{f0}\Psi_{s}\;U\tau \left(1-e^{-\frac{d_{f}}{U\tau }}\right)+I_{f,b}$$

where $\Psi_{s}$ is the weight function of U, and $I_{f,b}$ is the background fluorescence intensity when the dye is approximately fully photobleached. However, to simplify the analysis, they assumed the local fluorescence intensity ($I_{f,local}$) distribution in the focal area to be uniform, which could lead to deviation from the actual case, where $I_{f,local}$ normally has a Gaussian distribution. Moreover, the relationships among the complex fluorescent dynamics, the photobleaching reaction process and the superficial and measurable experimental parameters (e.g., τ) have not been established. This could be a major obstacle to the development of the LIFPA technique.

In this investigation, the photobleaching process is studied as a photochemical reaction, which can be further numerically analyzed by a convection–diffusion reaction process. We first established a relationship between the photobleaching time constant and photochemical reaction coefficient, by comparing experiments and numerical simulations, to reveal the relationships between the photobleaching reaction process and experimental parameters. Then, the influence of flow velocity and laser intensity on the effective concentration and fluorescent intensity was numerically investigated. At last, the spatial resolution of LIFPA was revisited to better understand the photobleaching phenomenon in flowing liquids and to develop new flow diagnostic techniques.

## 2. LIFPA Photobleaching Model

The photobleaching process of the fluorescent solution in a steady microchannel flow can be described by a convection–diffusion reaction equation [17,18,19] as

(3) $$\frac{\partial C}{\partial t}+\vec{u}\cdot \nabla C=D\nabla^{2}C+R_{c}$$

In Equation (3), C represents the concentration of the fluorescent solution, $\vec{u}$ is the flow velocity vector, D is the diffusivity of the dye molecule in the solution, and $R_{c}$ represents the reaction term according to photobleaching, which is determined by the laser intensity distribution, C and a factor $k_{b}$. By first-order approximation, $R_{c}$ can be expressed as

(4) $$R_{c}=-k_{b}IC$$

where $k_{b}$ represents the photobleaching rate of dye molecules per unit excitation intensity at a specific excitation wavelength, with a unit of cm^2^/(W·s). It represents how fast the fluorescent dye can be photobleached. The higher the $k_{b}$, the faster the photobleaching is. I is the intensity of the excitation laser; in general, I has a Gaussian distribution, shown as

(5) $$I=I_{max}G\left(x,y,z\right)$$

$I_{max}$ is the peak value of I, and G(x,y,z) is a 3D Gaussian function, shown as

(6) $$G\left(x,y,z\right)=\exp\left[-\left(\frac{{\left(x-x_{0}\right)}^{2}}{2\sigma_{x}{}^{2}}+\frac{{\left(y-y_{0}\right)}^{2}}{2\sigma_{y}{}^{2}}+\frac{{\left(z-z_{0}\right)}^{2}}{2\sigma_{z}{}^{2}}\right)\right]$$

where $\sigma_{x}$, $\sigma_{y}$ and $\sigma_{z}$ represent the standard deviations of laser intensity in x, y and z directions, respectively. Substituting Equations (4)–(6) into Equation (3), with dimensional analysis by

(7) $$C=C^{*}C_{0},x=x^{*}L,y=y^{*}L,\;z=z^{*}L,\vec{u}={\vec{u}}^{*}U_{0},\nabla =\nabla^{*}/L,\;t=t^{*}L/U_{0}$$

where $C_{0}$, L and $U_{0}$ are characteristic concentration, length scale and velocity, respectively, we have

(8) $$\frac{\partial C^{*}}{\partial t^{*}}+{\vec{u}}^{*}\cdot \nabla^{*}C^{*}=\frac{1}{P_{e}}\nabla^{*}{}^{2}C^{*}-\frac{k_{b}I_{\max}L}{U_{0}}G\left(x^{*},y^{*},z^{*}\right)C^{*}$$

where $P_{e}=U_{0}L/D$ is the Péclet number which represents the ratio of the convection rate to the diffusion rate. In microfluidic applications, normally the disturbance of microflow has the smallest time scale of O($10^{-4}$ s) or higher. In contrast, to obtain good sensitivity and resolution, LIFPA has a fast photobleaching process where τ is of O($10^{-6}$ s), which is approaching the lower limit of photobleaching lifetime, which is normally in the range of nanoseconds to seconds [20,21,22,23,24], or even longer. Meanwhile, the resident time of dye in the laser focus is O($10^{-4}$ s) or lower. Therefore, the time scale of flow is equivalent to or larger than the resident time and τ. A quasi-steady state of the photobleaching process of dye can be approximated in the laser focus region. Thus, $\partial C^{*}/\partial t^{*}=0$. The LIFPA photobleaching model under a quasi-steady state is obtained as

(9) $${\vec{u}}^{*}\cdot \nabla^{*}C^{*}=\frac{1}{P_{e}}\nabla^{*}{}^{2}C^{*}-Z_{pc}C^{*}G\left(x^{*},y^{*},z^{*}\right)$$

where $Z_{pc}=KL/U_{0}$ is a dimensionless number to evaluate the ratio between the photobleaching rate and convective transport rate of dye molecules. The larger the $Z_{pc}$, the stronger the local photobleaching is. Otherwise, the convective transport of the concentration of the fluorescent solution is more important. $K=k_{b}I_{\max}$ is the photochemical reaction coefficient, which represents the photobleaching rate of dye molecules in this investigation. It is a crucial parameter to characterize the photobleaching process through the convection–diffusion reaction equation in COMSOL simulation. Although K has a unit of 1/s, which is the same as 1/τ, K is not 1/τ. One of the focuses of this investigation is to find the relationship between K and τ. The characteristic concentration $C_{0}$ was eliminated during the simplification process. This indicates that increasing or decreasing the dye concentration C does not affect the first-order photobleaching process, if in the absence of light absorption. Finally, the local fluorescent intensity $I_{f,local}$ can be calculated accordingly as [25]

(10) $$I_{f,local}=q_{f}IC$$

with $q_{f}$ being a fluorescence quantum yield factor.

## 3. Experimental Setup

### 3.1. LIFPA System

**Figure 1:**
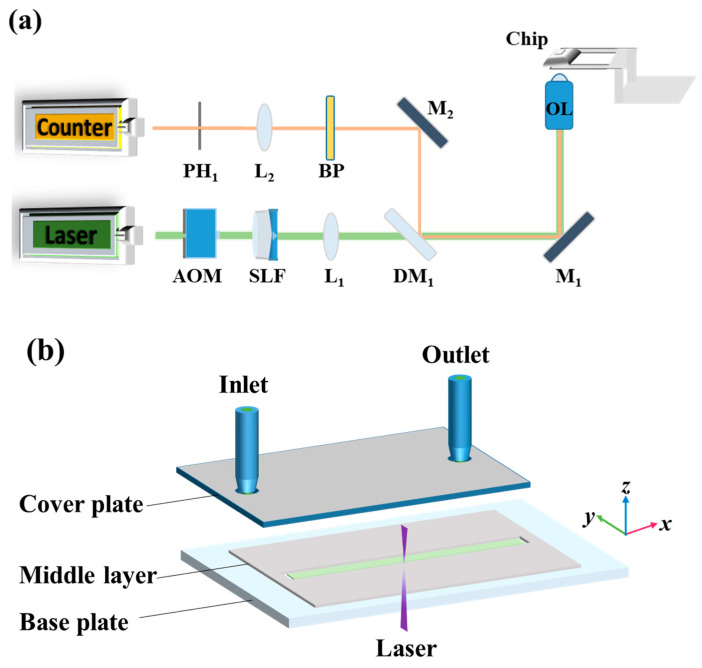
Experimental setup. (**a**) Schematic diagram of LIFPA experimental system based on confocal microscope; (**b**) schematic diagram of microchip.

The experimental LIFPA system is developed based on a confocal microscope, as shown in Figure 1a. The excitation laser is a 405 nm continuous wave laser (MDL-III-405-500, CNI). The excitation beam is first controlled by an acousto-optic modulator (AOM, 1206C-2-1002, Isomet) for on–off switch. Then, it passes through a spatial pinhole filter (SLF, SFB-16DM, OptoSigma) to improve the beam quality. A diaphragm is used to filter out the zero-order and high-order diffraction spots and retain the first-order spot in the center. After passing through a collimation lens, a collimated excitation beam with a Gaussian beam profile is obtained.

Subsequently, the excitation beam passes through the dichroic mirror (DM_1_), which transmits the 405 nm laser and reflects the fluorescence around 480 nm. The excitation beam is then reflected into the objective lens (OL, Olympus PlanApo, 100 × NA 1.4 oil immersion Objective lens) by a mirror (M_1_). The excitation beam is finally focused in the microchannel flow and illuminates the fluorescent dye. The fluorescent signal passes the optical path along M_1_, DM_1_, and a second mirror (M_2_), and then passes through a band-pass filter (BP, 470/10 nm, OptoSigma) to eliminate background light noise. The fluorescent signal is collected by a lens (L_2_) and focused on a pinhole PH_1_ for spatial filtering. The filtered fluorescent signal is detected by a photon counter (H7421-40, Hamamatsu). The exposure time of fluorescent measurement, i.e., the sampling duration, is 0.5 ms. A time interval of 0.5 ms is applied between two samples. Therefore, the sampling rate is 1 kHz. Since the photo counter has a saturation count rate of $1.5\times 10^{6}$ photons per second for linear measurement (equivalent to a maximum of 750 photons in the 0.5 ms sampling duration), and the fluorescence of LIFPA is not weak, we used a neutral density filter of OD2 in front of PH_1_, to reduce the fluorescent intensity and guarantee there is no nonlinear saturation of photon counter. In the experiments, the maximum photon number in a 0.5 ms sampling duration is below 533, which is in the linear range of the photon counter.

### 3.2. Microchannel and Solution Preparation

The experiment is carried out at the center of the microchannel. For precise positioning of the microchannel with a large travel distance, a 2D translation stage (PI M-521.DG, 1 μm accuracy) and a high-precision 3D piezo nanostage (PI 562.3CD, 1 nm accuracy) are applied together, as shown in Figure 1a. The structure of the microfluidic chip is shown in Figure 1b. It has three layers. The cover layer is made of 2 mm-thick acrylic with good transparency and mechanical strength. The middle layer, which is also the channel layer, is made of plastic sheets. The bottom plate is a low-fluorescence glass slide with a thickness of 130 μm. The chip is assembled layer-by-layer, to realize a microchannel with a rectangular cross section. The length, width, and height of the microchannel are 5 mm, 360 μm, and 90 μm, respectively. During experiments, the fluorescent solution of Coumarin 102 (C102, Sigma Aldrich) at a concentration of 0.1 mM is injected into the microchannel with a syringe pump to generate a laminar flow. The C102 solution is prepared by dissolving 2.5 mg of C102 powder with 95 mL of deionized water and 5 mL of methanol solution (Analytical Reagent, concentration > 99.5%). The C102 solution has a pH value of 7.82. Finally, after excitation, the photobleached fluorescent solution is drained from the outlet.

## 4. Numerical Simulation and Experiment

### 4.1. Numerical Simulation by COMSOL

**Figure 2:**
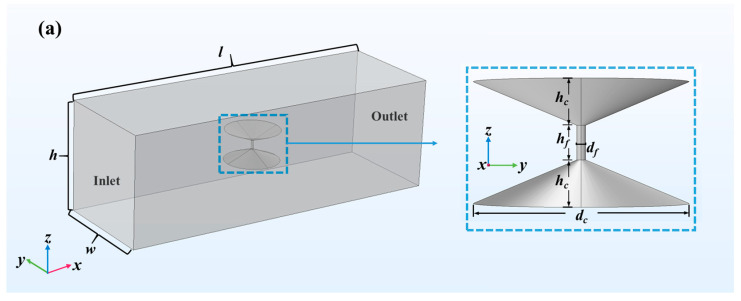

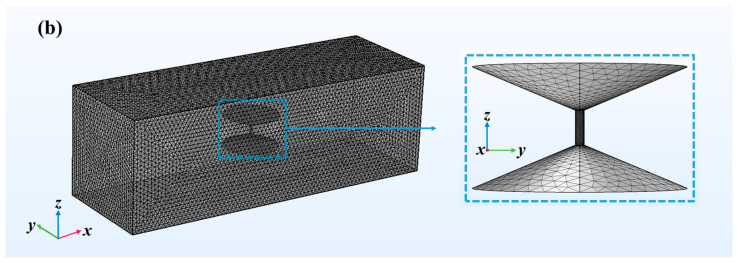
LIFPA simulation model in COMSOL. The coordinate origin is located at the geometric center. (**a**) Geometry of computation model of LIFPA. The inset shows the structural details of the laser focus region, which is also the reaction region; (**b**) details of mesh in COMSOL simulation.

In this paper, the photobleaching process of LIFPA is numerically investigated with COMSOL Multiphysics 4.3. The simulation is carried out in a 3D region with rectangular cross sections, as shown in Figure 2a.

A laser focus region approximately simulating the laser beam is located at the center (x=y=z=0) of the computational region and has an axisymmetric structure with 3 parts. At the middle part is a cylindrical region with diameter $d_{f}=203$ nm and height $h_{f}=800$ nm, whose diameter is coincident with the full width at half maximum (FWHM) of the laser beam. The top and bottom parts are symmetric cones with diameter $d_{c}\approx 5$ µm and height $h_{c}=1$ µm, respectively, to be coincident with the light field according to the high NA of the objective. In the 3D simulation, free tetrahedral elements with variable sizes are applied, as shown in Figure 2b. Since the size of the laser focus region of the excitation beam is only 203 nm, to have sufficient spatial resolution and smooth distribution of concentration field, the minimum size of the elements in the region is 0.5 nm. In the other regions, the minimum and maximum sizes of the elements are 100 nm and 530 nm, respectively. If we simulated the convection–diffusion reaction equation in the entire experimental microchannel, the computation burden is extraordinarily heavy. To save computation resources, in the simulation, we reduce the size of the computation region to 30 μm long, 10 μm wide and 10 μm high. The total number of free tetrahedral elements is 1,311,608.

The photobleaching process is simulated with laminar flow and dilute matter transfer modules in COMSOL. In the laminar flow module, the flow is assumed to be steady, uni-directional and fully developed. Therefore, we have $\vec{u}=U\hat{x}$ for simplification. A no-slip boundary condition [26,27] is been applied on the side walls (Ω) as

(11) $${U|}_{\Omega }=0$$

Since the sizes of the experimental microchannel are different from those of the computational region, to guarantee that the flow velocities at the laser focus regions of both experiments and simulations are the same, the flow rate in numerical simulation is different from that in experiments. In this investigation, we first calculate the flow velocity at the laser focus region (the third row of Table 1) through experimental flow rate (the first row of Table 1), according to Equation (12) [8,28,29]:

(12) $$Uy,z=\frac{48Q}{\pi^{3}wh}\left\{\overset{\infty }{\underset{n,odd}{\sum }}\frac{1}{n^{3}}\left[1-\frac{\cos h\left(\frac{n\pi y}{h}\right)}{\cos h\left(\frac{n\pi w}{2h}\right)}\right]\sin\left(\frac{n\pi z}{h}\right)\right\}{\left[1\;-\overset{\infty }{\underset{n,odd}{\sum }}\frac{192h}{n^{5}\pi^{5}w}\tan h\left(\frac{n\pi w}{2h}\right)\right]}^{-1}$$

where Q is the flow rate. −w/2≤y≤w/2 and −h/2≤z≤h/2. Then, the inlet flow rate in the simulation (the second row of Table 1) is calculated based on the flow velocity at the laser focus region (the third row of Table 1) in simulation, which is the same as in experiments. The outlet pressure is the circumstantial pressure. The flow is incompressible with a constant temperature.

In the dilute matter transfer module, the diffusion coefficient of molecules in the solution is $D=1\times 10^{-9}$ m^2^/s. The initial fluorescent solution concentration C=0.1 mM. At the side walls, no flux is present. The photobleaching of fluorescent molecules occurs only in the laser focus region in Figure 2. The photochemical reaction is stimulated by applying a reaction term based on Equations (4)–(6).

### 4.2. Direct Comparison between Experiments and Numerical Simulations

**Figure 3:**
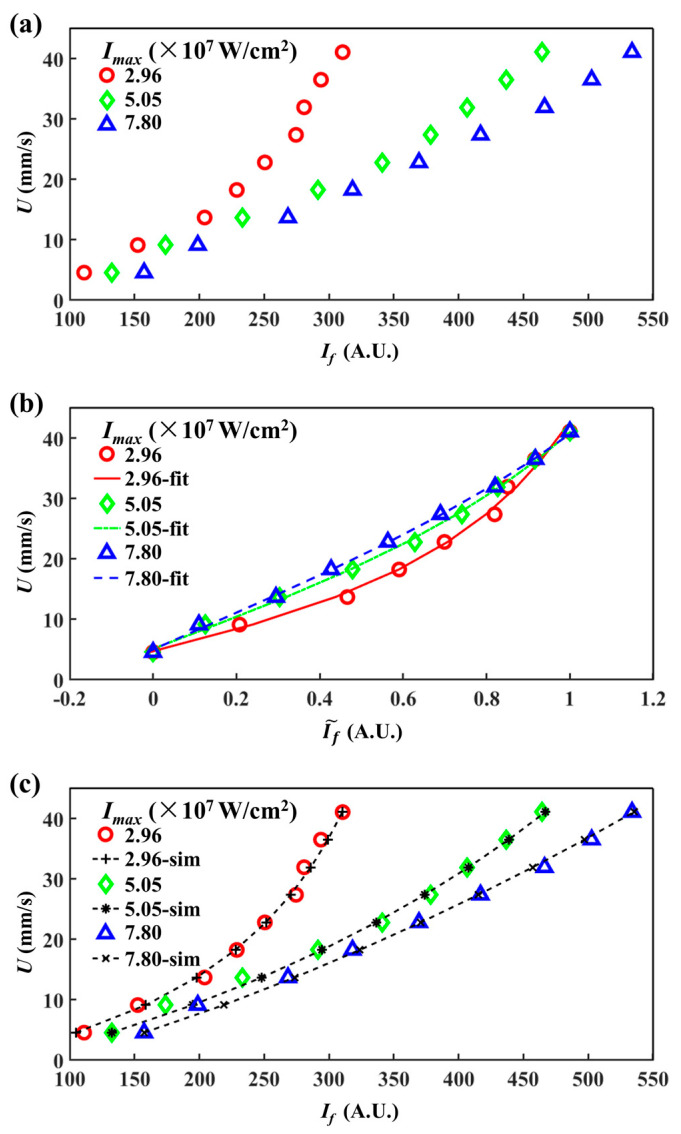

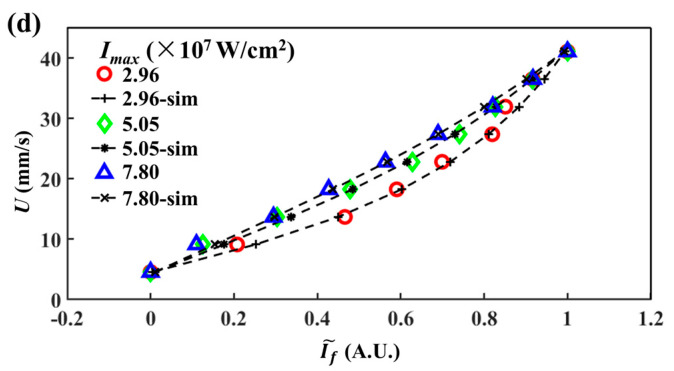
LIFPA velocity calibration curves. ‘fit’ and ‘sim’ represent the fitting and simulation calibration curves, respectively. (**a**) Experimental calibration curves for different laser power densities at center of the microchannel; (**b**) normalization for (**a**); (**c**) comparison of experimental and simulated calibration curves; (**d**) normalization for (**c**).

The relationships between U and fluorescence are established by experiments at three different laser powers (P) of 6.9, 11.8 and 18.2 mW. The corresponding $I_{\max}$ are $2.96\times 10^{7}$, $5.05\times 10^{7}$ and $7.80\times 10^{7}$ W/cm^2^, respectively, as shown in Figure 3a. Each data point was calculated by averaging over $2\times 10^{4}$ fluorescent signals, i.e., a 20 s time sequence of fluorescent signals. It is obvious that $I_{f}$ increases with the increasing U. Moreover, when the laser power density is small (e.g., $I_{\max}=2.96\times 10^{7}$ W/cm^2^), $I_{f}$ shows an obvious nonlinear increment with U. When $I_{\max}$ is increased to $5.05\times 10^{7}$ and $7.80\times 10^{7}$ W/cm^2^, the nonlinear behavior gradually decreases and the curve becomes more linear, accompanied by a decreasing slope of the $U\sim \tilde{I_{f}}$ curve. In these cases, velocity fluctuations can be more clearly distinguished by larger fluorescent fluctuations, as shown in Figure 3a. Thus, high laser power density can provide a better sensitivity of LIFPA measurement.

In order to compare $U\sim \tilde{I_{f}}$ relationships at different laser power densities, we calculate the normalized fluorescence intensity ($\tilde{I_{f}}$) with the maximum ($I_{f,\max}$) and minimum ($I_{f,\min}$) values as [2]

(13) $$\tilde{I_{f}}=\frac{I_{f}-I_{f,\min}}{I_{f,\max}-I_{f,\min}}$$

The $U\sim \tilde{I_{f}}$ curves are shown in Figure 3b. It can be clearly seen that the lower the laser power density of the excitation laser, the stronger the curve bending. After normalization, the $U\sim \tilde{I_{f}}$ curves under each $I_{\max}$ only lie in the different bending. In the experiments, we use the photobleaching time constant (τ) to evaluate the strength of the photobleaching. τ can be approximately calculated by nonlinear fitting on $U\sim \tilde{I_{f}}$ through the equation below [2]

(14) $$\tilde{I_{f}}=aU\left[1-\exp\left(-\frac{d_{f}}{U\tau }\right)\right]+b$$

where a is an amplification factor which dominates the slope of the $U\sim \tilde{I_{f}}$ curve and b represents the initial fluorescence intensity of $I_{f}$ at U=0. τ determines the curvature of the curve. According to Equation (14), a set of $U\sim \tilde{I_{f}}$ curves are nonlinearly fitted (as shown in Figure 3b) to calculate the corresponding τ. The results of τ calculated from the experiments are listed in Table 2. It can be seen, as P is increased, that τ apparently decreases to as low as 2.43 μs.

**Figure 4:**
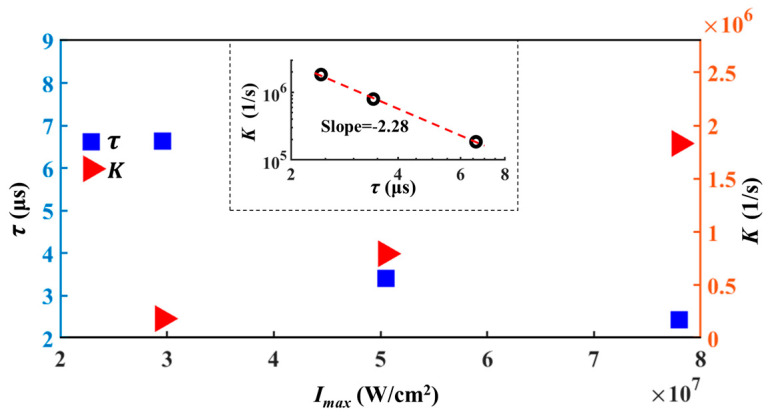
Comparisons among photochemical reaction coefficient K, photobleaching time constant τ and laser power density $I_{max}$. The inset displays the relationship of K and τ in a log–log plot.

Subsequently, the numerically calculated $U\sim \tilde{I_{f}}$ curve is compared with that of the experiments. In the simulation, $I_{f}=\int \int \int_{D}I_{f,\mathrm{local}}dV$, where D is a cylindrical integration region (i.e., detection region) for fluorescent collection, with a height of $h_{f}$ and diameter of $d_{cl}$. In this section, D is the cylindrical part of the inset of Figure 2a and $d_{cl}=d_{f}$, to be coincident with the experimental system we applied. As shown in Figure 3c,d, both the original and normalized numerically calculated fluorescence curves show high consistency with the experimental ones. Since the numerical calculated $U\sim \tilde{I_{f}}$ curves are merely determined by the photochemical reaction coefficient K, accordingly, a relationship between τ and K can be established, as shown in Figure 4 and Table 2. When $I_{\max}$ is increased, we find K increases with $I_{\max}$ in an approximately linear manner, while K decreases with τ as $K\sim \tau^{-2.28}$, as shown in the inset of Figure 4.

This indicates that the fluorescent photobleaching time shows a nonlinear relationship with K under strong laser illumination. To achieve a higher temporal resolution with smaller τ, K should be significantly increased.

### 4.3. Effective Concentration Distribution

**Figure 5:**
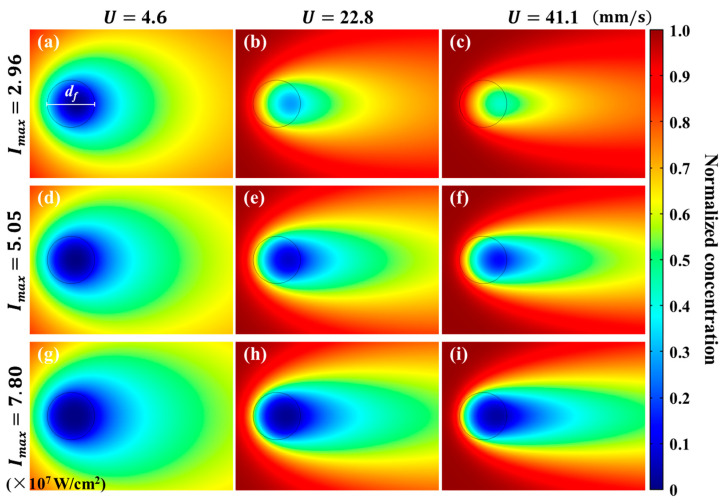
Distribution of effective concentration C of fluorescent dye under different bulk flow velocities and laser power densities. (**a**–**c**) C under $I_{\max}=2.96\times 10^{7}$ W/cm^2^, with U=4.6,  22.8,  41.1 mm/s, respectively. (**d**–**f**) C under $I_{\max}=5.05\times 10^{7}$ W/cm^2^, with U=4.6, 22.8, 41.1 mm/s, respectively. (**g**–**i**) under $I_{\max}=7.80\times 10^{7}$ W/cm^2^, with U=4.6, 22.8, 41.1 mm/s, respectively. Here, the concentration values have been normalized by the maximum C. The black circle represents the integration region of fluorescence to calculate $I_{f}$.

The influence of photobleaching on the effective concentration C of fluorescent dye can be directly observed in Figure 5. The photobleaching generates a cometlike region of C. When the flow velocity U at the focus region is small (e.g., U=4.6 mm/s), as shown in Figure 5a, a strongly photobleached region can be found in the focus region of the laser. The cometlike region of C has a large width attributed to the lateral diffusion. When U is increased, the fluorescent dye is less photobleached and C in the focus region is significantly higher than the counterparts of smaller U, as can be seen from Figure 5b,c. The tails of the cometlike regions exhibit larger length and smaller width. Downstream of the focus area, C gradually recovers due to the molecular diffusion. Similar results can also be found in the second and third rows of Figure 5.

The distribution of C is dominated by three dimensionless numbers, i.e., $P_{e}$ and $Z_{pc}$, in Equation (9), and $Z_{pd}=Z_{pc}P_{e}\;$, which is the ratio between photobleaching rate and diffusion rate. When $P_{e}$ is increased with constant $Z_{pc}$, the influence of diffusivity becomes smaller and the width of the cometlike region is decreased. When $Z_{pc}$ is increased with constant $P_{e}$, the influence of photobleaching is enhanced and the value of C becomes smaller in the focus area. When $Z_{pd}$ is fixed, the tail length of the cometlike region of C increases with $P_{e}$.

### 4.4. Fluorescence Intensity Distribution

**Figure 6:**
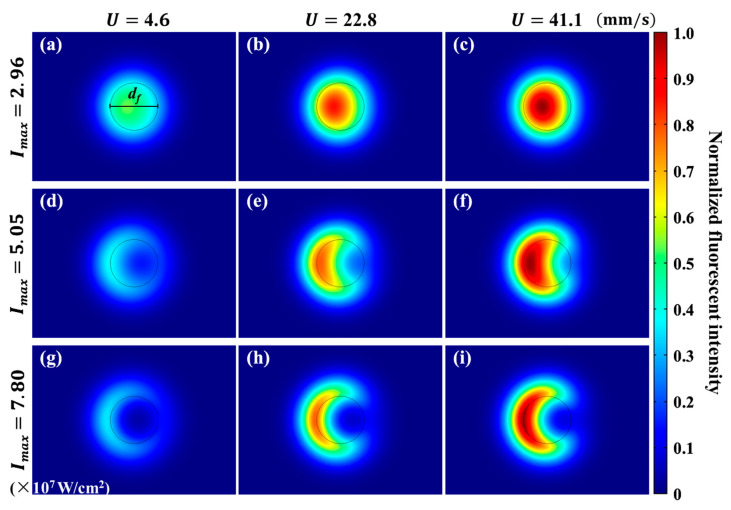
Distribution of fluorescent intensity $I_{f,\mathrm{local}}$ under different bulk flow velocities and laser power densities. (**a**–**c**) $I_{f,\mathrm{local}}$ under $I_{\max}=2.96\times 10^{7}$ W/cm^2^, with U=4.6, 22.8, 41.1 mm/s, respectively. (**d**–**f**) $I_{f,\mathrm{local}}$ under $I_{\max}=5.05\times 10^{7}$ W/cm^2^, with U=4.6, 22.8, 41.1 mm/s, respectively. (**g**–**i**) $I_{f,\mathrm{local}}$ under $I_{\max}=7.80\times 10^{7}$ W/cm^2^, with U=4.6, 22.8, 41.1 mm/s, respectively. Here, the legend is normalized by the peak fluorescent intensity. The black circle represents the integration region of fluorescence to calculate $I_{f}$.

The distributions of fluorescence under different flow velocities and excited by different laser power densities are calculated according to Equation (10). The results are shown in Figure 6, where the fluorescent intensity is normalized by the maximum value. It is obvious that the fluorescent intensity in the focus area is symmetric on the *x* axis, and decreases along the flow direction as expected.

As shown in Figure 6a–c, when U is increased with fixed $I_{\max}=2.96\times 10^{7}$ W/cm^2^, $P_{e}$ is increased, while $Z_{pc}$ is decreased. The streamwise positions of the peak fluorescent intensities move downstream and toward the center of the focus area. Similar results can also be found on the second and third rows. When $I_{\max}$ is increased with fixed U=41.1 mm/s, $P_{e}$ is constant, while $Z_{pc}$ is increased. From Figure 6c,f,i as examples, it can be seen that the streamwise positions of the peak fluorescent intensities move upstream and even leave the integration region. The fluorescent intensity at the center of focus area continuously decreases.

## 5. Velocity Measurement of Breaking Optical Diffraction Limit

Figure 6d,g indicate, as $Z_{pc}$ is increased, the major body of fluorescence moves upstream and even out of the integration region. This could lead to a “waste” of fluorescence for calculating flow velocity. This reminds us to rethink the influence of the integration region on the performance, especially the spatial resolution of the LIFPA system. Therefore, we revisited the spatial resolution of LIFPA first in this section. Then, we discuss a possible method to break the optical diffraction limit and realize a super-resolution velocity measurement. 

### 5.1. Spatial Resolution of Effective Velocity Measurement with LIFPA

**Figure 7:**
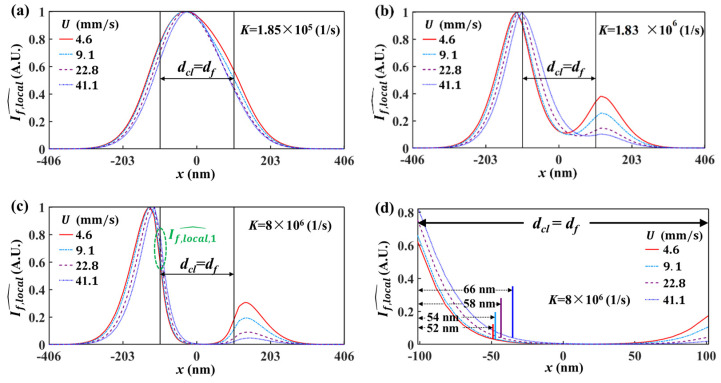
$\hat{I_{f,\mathrm{local}}}\sim x$ curves normalized at different U under different K. Here, $d_{cl}=d_{f}=203$ nm. In each figure, the fluorescent intensity profiles are plotted with four different U from 4.6 mm/s to 41.1 mm/s. (**a**) $K=1.85\times 10^{5}$ 1/s, (**b**) $K=1.83\times 10^{6}$ 1/s, (**c**) $K=8.00\times 10^{6}$ 1/s, (**d**) zoomed-in view of the integration region in (**c**).

To investigate the details of the photobleaching process of LIFPA, we calculated the fluorescent intensity profile $\hat{I_{f,\mathrm{local}}}$ ($\hat{I_{f,\mathrm{local}}}=I_{f,\mathrm{local}}/\underset{y=z=0}{\max}\left(I_{f,\mathrm{local}}\right)$) through the center of the laser focus spot (−406≤x≤406 nm), as shown in Figure 7. It can be seen, when $K=1.85\times 10^{5}$ 1/s (Figure 7a), the $\hat{I_{f,\mathrm{local}}}$ curves according to different U show a small difference. As K is further increased, as shown in Figure 7b,c, the fluorescent intensity at the center part becomes smaller and the streamwise positions of the peak $\hat{I_{f,\mathrm{local}}}$ move downstream at larger U. One noteworthy phenomenon is that when K is sufficiently large (see Figure 7c,d as an example), there is a valley with flat $\hat{I_{f,\mathrm{local}}}$, where the fluorescent dye molecules have been strongly photobleached. When measuring velocity fluctuations, only the part marked with a width of $d_{\mathrm{eff}}$ (i.e., actual resolution, which is defined as the distance from the left edge of the integration region to the position where $\hat{I_{f,\mathrm{local}}}=0.05\hat{I_{f,\mathrm{local},1}}$, $\hat{I_{f,\mathrm{local},1}}$ is marked in Figure 7c and represents the $\hat{I_{f,\mathrm{local}}}$ at the left edge of the integration region) in Figure 7d has an apparent contribution to the fluorescent variation. The other part in the integration region has a negligible contribution to the fluorescent variation, and accordingly, the velocity variation, especially at higher velocity. In other words, the velocity measurement by LIFPA is only sensitive in the marked part, which can be considered as the point spreading function (PSF) of the fluorescent intensity. Therefore, the width of PSF, i.e., $d_{\mathrm{eff}}$, actually determines the spatial resolution of LIFPA.

This is intriguing, since in conventional LIFPA techniques, the spatial resolution of LIFPA is believed to be equivalent to the spatial resolution of the optical system (i.e., the FWHM of the point spread function). From the current research, it can be seen when K is sufficiently large, e.g., $K=8\times 10^{6}$ 1/s, the averaged $d_{\mathrm{eff}}$ over the four curves shown in Figure 7d is around 58 nm. The actual spatial resolution of LIFPA can be apparently smaller than that of the optical system (203 nm in this investigation) and the diffraction limit, i.e., the super-resolution of velocity measurement can be realized. Specifically, this can be achieved by simply increasing $I_{\max}$ in a confocal microscope, or properly selecting a fluorescent dye with large $k_{b}$. For instance, if $K=I_{\max}k_{b}$ is increased to $2\times 10^{7}$ 1/s, the averaged $d_{\mathrm{eff}}$ can theoretically be as low as 30 nm.

Relative to the LIFPA system, developed on the basis of stimulated emission depletion (STED) super-resolution techniques [6] to realize super-resolution velocity measurements, where two beams (one excitation beam and one depletion beam) must be collimated and well aligned, the current investigation shows that LIFPA can be intrinsically super-resolution even with a simple confocal microscope, if K is sufficiently large. Additionally, in the STED LIFPA system, the power density of the excitation beam cannot be very high, otherwise the spatial resolution of STED is reduced. Thus, the temporal resolution of the STED LIFPA system is normally low. However, for a sufficiently large K, both ultrahigh spatial and temporal resolutions can be achieved simultaneously.

### 5.2. Influence of Integration Region on Velocity Measurement

**Figure 8:**
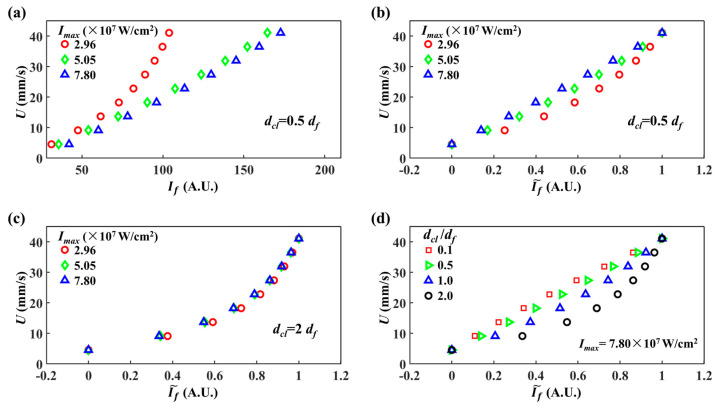
LIFPA velocity calibration curves. (**a**) Original calibration curves for different laser power densities at $d_{cl}=0.5d_{f}$; (**b**) normalized calibration curves for (**a**); (**c**) normalized calibration curves for different power densities at $d_{cl}=2d_{f}$; (**d**) normalized calibration curves for different $d_{cl}$ at $I_{\max}=7.80\times 10^{7}$ W/cm^2^.

In this section, we further study the influence of $d_{cl}$ of integration region on the velocity calibration curve, and attempt to show another way to improve LIFPA’s spatial resolution. The results are plotted in Figure 8. Relative to Figure 3a where $d_{cl}=d_{f}$, the velocity calibration curves calculated at $d_{cl}=0.5d_{f}$ show clearly smaller values and the value ranges of $I_{f}$, as plotted in Figure 8a, in line with our expectations. Although it could lead to a negative influence on velocity measurement due to worse signal-to-noise ratio (SNR), the spatial resolution could be clearly improved to super-resolution level. From Figure 3b and Figure 8b,c, it can be seen that changing $d_{cl}$ will not affect the result that the curves show more bending at lower $I_{\max}$. As $d_{cl}$ is increased, the influence of $I_{\max}$ on the bending of velocity calibration curves becomes smaller, as can be seen from Figure 8b,c. The $U\sim \tilde{I_{f}}$ plots become indistinguishable between $I_{\max}=5.05\times 10^{7}$ and $7.80\times 10^{7}$ W/cm^2^ in Figure 8c. We plot four $U\sim \tilde{I_{f}}$ curves of different $d_{cl}$ in Figure 8d at $I_{\max}=7.80\times 10^{7}$ W/cm^2^. Despite the smaller fluorescent signal at smaller $d_{cl}$, the velocity calibration curve becomes more linear at smaller $d_{cl}$. It again indicates the temporal resolution is also improved, accompanied by the spatial resolution.

**Figure 9:**
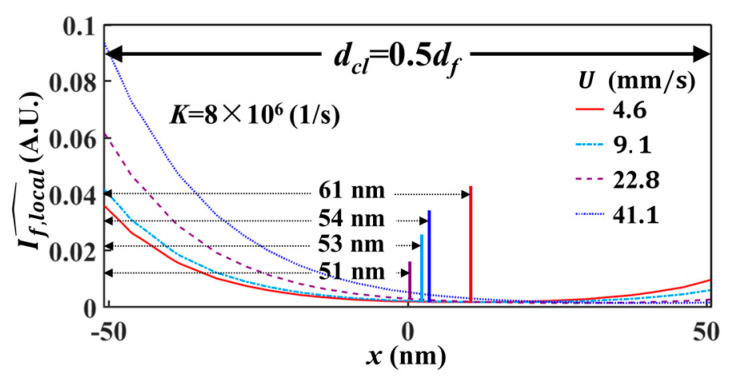
Zoomed-in view of the integration region in the $\hat{I_{f,\mathrm{local}}}\sim x$ curves normalized at different U, if $d_{cl}=0.5d_{f}$ and $K=8.00\times 10^{6}$ 1/s.

In addition, we plotted the $\hat{I_{f,\mathrm{local}}}\sim x$ under $d_{cl}=0.5d_{f}$ in Figure 9. It can be seen that as $d_{cl}$ is reduced, the corresponding $d_{\mathrm{eff}}$ is also reduced. At $K=8.00\times 10^{6}$ 1/s, the averaged $d_{\mathrm{eff}}$ over the four curves is around 55 nm, which is slightly smaller than that at $d_{cl}=d_{f}$ shown in Figure 7d. Thus, by decreasing the size of the integration region, the actual spatial resolution of LIFPA can be further enhanced.

## 6. Conclusions

In this investigation, we studied the photobleaching process of fluorescent dye in the laser focus region, according to the model based on the convection–diffusion reaction equation. The profiles of effective dye concentration and fluorescence were elucidated. The relationship between the commonly used photobleaching time constant obtained by experiments and the photochemical reaction coefficient was revealed. We further studied the influence of the detection region of fluorescence on the performance of the LIFPA system, and found that at sufficiently high excitation laser power density, LIFPA can achieve super-resolution and break the limit of optical diffraction, even in a confocal microscope. We hope the current investigation can promote the development of LIFPA, and reveal the photobleaching process of fluorescent dye under high laser power density illumination, to enhance our understanding of fluorescent dynamics and photochemistry and develop more powerful photobleaching-related flow diagnostic techniques.

## Figures and Tables

**Table 1:** Flow conditions in experiments and numerical simulations.

Flow Rate in experiments (μL/min)	5.0	10.0	15.0	20.0	25.0	30.0	35.0	40.0	45.0
Flow rate in simulation (nL/min)	13.1	26.1	39.2	52.2	65.3	78.3	91.4	104.5	117.5
U(0,0) for both experiments and simulations (mm/s)	4.6	9.1	13.7	18.2	22.8	27.4	31.9	36.5	41.1

**Table 2:** Experimental results of τ, peak I (say $I_{\max}$) and the corresponding photochemical reaction coefficient K in numerical simulation under different P.

P (mW)	6.90	1.80	18.20
$I_{max}$ (W/cm^2^)	2.96 × 10^7^	5.05 × 10^7^	7.80 × 10^7^
τ (μs)	6.63	3.41	2.43
K (1/s)	1.85 × 10^5^	7.95 × 10^5^	1.83 × 10^6^
